# Osteoprotective Effect of Echinocystic Acid, a Triterpone Component from *Eclipta prostrata*, in Ovariectomy-Induced Osteoporotic Rats

**DOI:** 10.1371/journal.pone.0136572

**Published:** 2015-08-28

**Authors:** Ya-ting Deng, Wen-bo Kang, Jian-ning Zhao, Gang Liu, Ming-gao Zhao

**Affiliations:** 1 Department of Pharmacology, School of Pharmacy, Fourth Military Medical University, Xi’an, China; 2 Department of Orthopedics, Jinling Hospital, Clinical School of Nanjing, Second Military Medical University, Nanjing, China; Université de Lyon—Université Jean Monnet, FRANCE

## Abstract

Echinocystic acid (EA) is a natural triterpone enriched in various herbs and has been used for medicinal purposes in China. In the present study, we systematically examined the effects of EA on ovariectomy-induced osteoporosis in rats for the first time. Three-month-old female ovariectomy (OVX) Sprague–Dawley rats were used to evaluate the osteoprotective effect of EA. Results showed that administration of EA (5 or 15 mg/kg/day) for 12 weeks prevented lower levels of maximum stress and Young’s modulus of femur induced by OVX. EA also recovered bone metabolic biomarkers levels in OVX rats, including osteocalcin, alkaline phosphatese, deoxypyridinoline, and urinary calcium and phosphorus. EA (5 and 15 mg/kg/day) could prevent the alteration of total bone mineral density in the femur caused by OVX. However, only high dose (15 mg/kg/day) of EA significantly improved trabecular architecture, as evidenced by higher levels of bone volume/tissue volume, trabecula number, and trabecula thickness, and lower levels of trabecula separation and structure model index compared with OVX rats. In addition, EA treatment decresed the serum levels of IL-1β and TNF-α in OVX rats. In conclusion, EA could prevent reduction of bone mass and strength and improve the cancellous bone structure and biochemical properties in OVX rats. Hence, EA may serve as a new candidate or a leading compound for anti-osteoporosis.

## Introduction

Osteoporosis, characterized by low bone mineral density (BMD) and fragility of the bone, is a worldwide health problem for the aging population. Approximately 40 percent of women over the age of 50 suffer from osteoporotic fracture[1]. Recent studies have suggested that pro-inflammatory cytokines (IL-1β, IL-6, IL-8, TNF-α) play an important role in postmenopausal osteoporosis[2,3].

Present drugs offer specific and clearly targeted therapeutic effects on the improvement of bone quality; however, side effects remain the clinical problems for patients[4,5,6] In recent years, there is a growing interest in developing anti-osteoporosis agents from plants. Flavonoids derived from food and herbs have been shown to have anti-osteoporotic effects in animal models and clinical trials [7,8,9,10,11,12]. However, other kinds of phytochemicals received less attention for their potential role in preventing osteopenia. Recently, several triterpenoids, including oleanolic acid, ursolic acid, and their derivatives, have been reported to prevent bone loss by stimulating the osteoblastic differentiation of bone mesenchymal stem cells and inhibiting the formation of osteoclasts [13,14,15].

Echinocystic acid (EA) is one of the triterpenes sharing a similar structure with oleanolic acid. Multiple pharmacological effects of EA have been found including anti-inflammation [16,17,18], anti-viral [19], cytotoxic activity to cancer cells [20], and protective effects against acute myocardial ischemia [21]. EA is a natural triterpone enriched in various herbs, including *Eclipta prostrata*. A previous study has demonstrated that *Eclipta prostrate* has beneficial effects on ovariectomy (OVX)-induced osteoporotic rats [22]. Based on the above researches, it is reasonable to hypothesize that EA might take a active role in estrogen defciency-related osteoporosis. However, little is known on the anti-osteoporotic effects of EA on bone loss. Therefore, the purpose of this study is to evaluate the effects of EA on osteoporosis induced by OVX in rats, and it has been found that EA reversed the reduction of bone mass and strength and improved the cancellous bone structure and biochemical properties in OVX rats.

## Materials and Methods

### Drugs and reagents

17β-estradiol (E2) was purchased from the Xi’an Hualian Pharmaceutical. The kits for measurement of calcium, inorganic phosphorus and alkaline phosphatase activity were obtained from ZhongSheng BeiKong Bio-technology and Science (Beijing, China). Enzyme-Linked Immuno Sorbent Assay (ELISA) kits for measurement of deoxypyridinoline (DPD), osteocalcin (also called bone Gla Protein, BGP), IL-1β and TNF-α were purchased from Elabscience Biotechnology Co., Ltd (Wuhan, China).

### Animals and treatments

Three-month-old female Sprague–Dawley rats (220–250 g) were purchased from the Experimental Animal Center of the Fourth Military Medical University. Rats were given standard food and water per day under controlled conditions of temperature (23 ± 2°C) and relative humidity (50% ± 5%) and light-dark cycle (light on from 8 a.m. to 8 p.m.) throughout the experimental period [23]. After a 1-week adaptation period, 10 rats were sham-operated and treated with vehicle as aging control (sham). The remaining rats were bilaterally ovariectomized and randomly divided into five groups with 10 rats per group. The operation was performed under pentobarbital sodium (35 mg/kg, i.p.) anesthesia. After operation rats were kept at the incubator to maintain body temperature until they recovered; the weight and daily food consumption were monitored in the following weeks. Four weeks after surgery, the OVX rats were treated with vehicle, E2 (25 μg/kg/day, ig), or EA (1, 5, and 15 mg/kg/day, ig) for 12 weeks. Body weight was recorded once a week throughout the experiment. There were no any unintended deaths during this study. To minimize the suffering of the animals, animals were first anesthetized with 2.5% isoflurane before sacrifice. Before anesthesia, urine samples were collected individually for 24 h in metabolic cages without providing food. Blood samples were taken from abdominal aorta under isoflurane anesthesia. Uteri were removed and immediately weighed. Femurs were dissected and stored in physiological saline at −80°C until examination for biomechanical testing and structural analysis. This study was approved by the institutional ethics committee of the Fourth Military Medical University.

### Measurements of bone biomechanical strength

The mechanical properties of the left femurs were determined by a three-point bending test [24,25]. The biomechanical parameters of the left femoral diaphysis was measured by a CMT4204 material testing machine (Shenzhen Skyan Power Equipment Co. Ltd., Shenzhen, China). Briefly, the left femurs were thawed at room temperature for at least 1 h, and then placed in the material test machine with 20 mm distance between two support points. The loading speed was set at 2 mm/min. The ultimate load (maximum load), energy absorbed to failure, elastic modulus and ultimate stress (maximum stress) were obtained, and the bone load-displacement curves were plotted with the software simultaneously [25].

### Assay for serum and urine chemistry

Serum calcium (S-Ca), phosphorus (S-P), and alkaline phosphatase (ALP) concentrations were measured by commercial kits and analyzed by a Cobas Integra 400 Plus automatic biochemical analyzer (Roche Diagnostics, Basel, Switzerland). Urine calcium (U-Ca), phosphorus (U-P), and creatinine (Cr) concentrations were evaluated by the same method. Serum levels of osteocalcin (OC),IL-1β and TNF-α were determined by ELISA kits. Urinary deoxypyridinoline (DPD) level was also assayed using ELISA kit. The urinary excretion of Ca and DPD were expressed as the ratio to Cr concentration (Ca/Cr and DPD/Cr) [25].

### Bone microarchitecture assessment by micro-computed tomography

Bone microarchitecture in distal femur was scanned by eXplore Locus SP Pre-Clinical Specimen Micro-Computed Tomography (GE Healthcare, USA). The reconstruction and 3D quantitative analyses were performed by the desktop micro-computed tomography system. In the femora, the scanning regions were confined to the distal metaphysis, extending proximally 2.0 mm from the proximal tip of the primary spongiosa. The following 3D indices in the defined region of interest were analyzed, including relative bone volume over total volume (BV/TV, %), trabecular separation (Tb.Sp), trabecular number (Tb.N), trabecular thickness (Tb.Th), structure model index (SMI) and BMD[25,26]. The operator performing the scan analysis was blinded to the treatments revelant with the specimens.

### Statistical analysis

Results were expressed as mean ± SEM. Data were evaluated using one-way analysis of variance (ANOVA) for *post hoc* comparisons (SPSS 13.0). The data that passed the homogeneity test were analyzed by the one-way ANOVA Least Significant Difference (LSD) test. Data that did not pass the homogeneity test were analyzed by the one-way ANOVA Dunnett’s T3 test [27]. In all cases, *p <* 0.05 was considered statistically significant.

## Results

### EA had no effects on body and uterine weight in OVX rats

**Fig 1:**
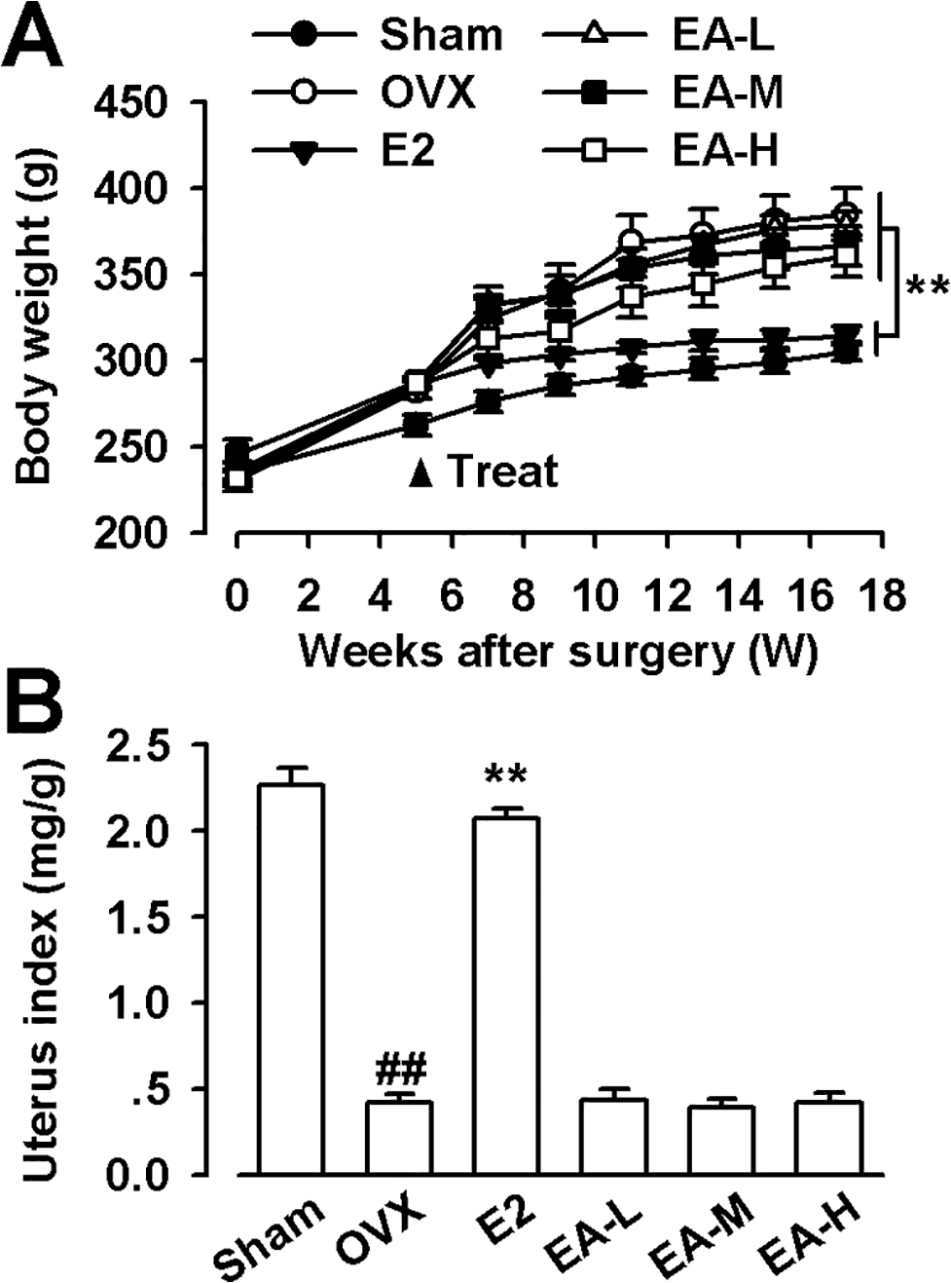
Effects of EA on body weight and uterus index. **(A)** Effects of E2 and EA on body weight. **(B)** The uterus index was represented as uterus weight divided by body weight. E2 (25 μg/kg/day); EA-L (1 mg/kg/day); EA-M (5 mg/kg/day); EA-H (15 mg/kg/day). n = 10; ** *p* < 0.01 vs OVX; ^##^ *p* < 0.01 vs Sham.

The body weight of OVX rats was markedly increased from the 5^th^ week after operation compared with the sham rats. E2 treatment could prevent increase in body weight, whereas EA (1, 5, and 15 mg/kg/day) had no effects on the body weight in OVX rats (Fig 1A). As predicted, the uterine weight of OVX significantly decreased, indicating the success of the surgical procedure. E2 treatment could prevent the decrease of uterine weight, whereas EA did not affect the uterine weight in the OVX rats (F_(5, 54)_ = 19.245, *p* < 0.01, Dunnett’s T3 test; *p* = 0.001, OVX vs. Sham; *p* = 0.001, E2 vs OVX; Fig 1B).

### EA improves biomechanical properties of bone

Three-point bending test was performed in the left femur to determine the bone strength. The extrinsic biomechanical property of the bone was evaluated by ultimate load and energy absorbed to failure, while elastic modulus and ultimate stress were assayed as intrinsic biomechanical parameters. As shown in Table 1, OVX induced a slight decrease in the levels of the maximum load and energy, but resulted in a significant decrease in the elastic modulus and maximum stress (F_(5, 54)_ = 16.319, *p* < 0.01, Dunnett’s T3 test; *p* = 0.000, OVX vs Sham for elastic modulus; F_(5, 54)_ = 2.827, *p* > 0.01, LSD test; *p* = 0.005, OVX vs Sham for maximum stress).The E2 and EA administration significantly prevented the decrease of elastic modulus induced by OVX (*p* = 0.009, E2 vs OVX; *p* = 0.006, EA-M vs OVX; *p* = 0.000, EA-H vs OVX; Table 1). As predicted, maximum stress was also significantly higher compared with the OVX group after EA and E2 treatment (*p* = 0.046, E2 vs OVX; *p* = 0.049, EA-M vs OVX; *p* = 0.045, EA-H vs OVX; Table 1). Lower dose of EA (1 mg/kg) had marginal effects on the biomechanical properties (*p* = 0.362, EA-L vs OVX for elastic modulus; *p* = 0.139, EA-L vs OVX for maximum stress; Table 1).

**Table 1:** Effects of EA on bone biomechanical parameters in femoral diaphysis of OVX rats.

Parameters	Sham	OVX	E2	EA-L	EA-M	EA-H
Elastic modulus	6336±203	4679±112^##^	6123±227**	5100±359	5844±254*	6374±157**
(MPa)						
Maximum stress	165±18	137±8^##^	152±3**	137±3	150±1*	155±20**
(MPa)						
Maximum load	125±5	111±5	113±3	123±13	123±8	123±9
(N)						
Energy	54±1	51±1	54±1	51±1	51±1	54±19
(N*mm)						

EA-L: EA (1 mg/kg); EA-M: EA (5 mg/kg); EA-H: EA (15 mg/kg); n = 10

*p < 0.05

**p < 0.01 vs OVX

^##^ *p* < 0.01 vs sham.

### EA prevented alterations of bone metabolic biomarkers in OVX rats

**Fig 2:**
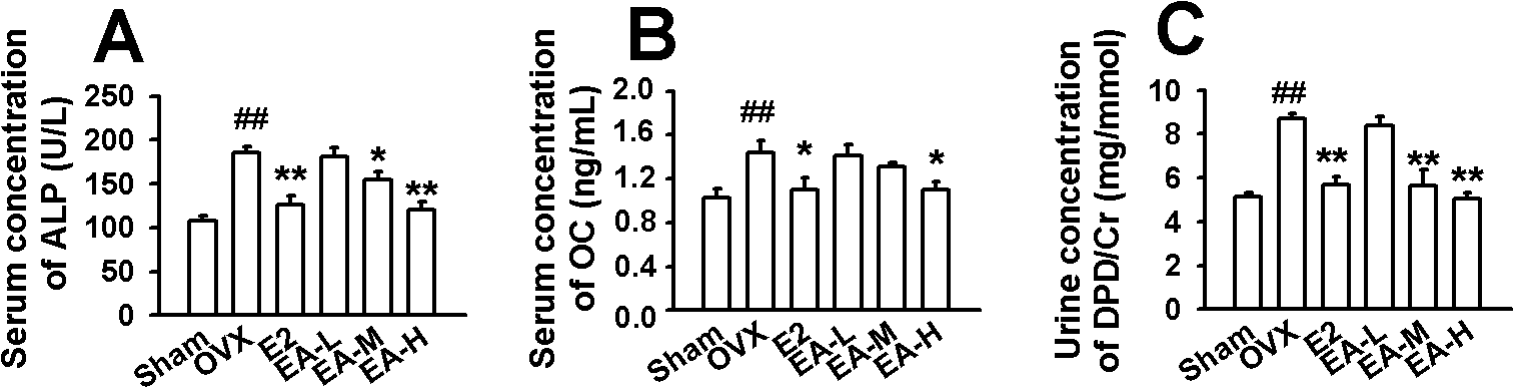
Effects of EA on bone formation and resorption parameters. **(A)** Effects of E2 and EA on serum alkaline phosphatase (ALP). **(B)** Effects of E2 and EA on serum osteocalcin (OC). **(C)** Effects of E2 and EA on urinary deoxypyridinoline (DPD/Cr). E2 (25 μg/kg/day); EA-L (1 mg/kg/day); EA-M (5 mg/kg/day), EA-H (15 mg/kg/day). n = 10; * *p* < 0.05, ** *p* < 0.01 vs OVX; ^##^ *p* < 0.01 vs Sham.

In OVX group, the U-Ca and U-P levels were higher compared with the sham group; however, the levels of S-Ca and S-P did not show changes among the groups (Table 2). Treatment with EA or E2 reversed the elevated U-Ca levels in OVX rats (F_(5, 54)_ = 18.680, *p* < 0.01, Dunnett’s T3 test; *p* = 0.000, E2 vs OVX; *p* = 1.000, EA-L vs OVX; *p* = 0.002, EA-M vs OVX; *p* = 0.000, EA-H vs OVX). Only high dose of EA (15 mg/kg/day) recovered the U-P levels in OVX rats (F_(5, 54)_ = 1.068, *p* > 0.01, LSD test; *p* = 0.032, EA-H vs OVX; Table 2). The serum ALP level is an early phase marker of osteoblast hyperactivity and bone formation during the matrix maturation phase [28,29]. Osteocalcin, one of the major non-collagenous proteins secreted solely by osteoblasts, is a late phase osteoblastic differentiation marker[30,31]. OVX induced higher levels of serum ALP and osteocalcin sixteen weeks after operation. These alterations were recovered by treatment with EA or E2 in the OVX rats (F_(5, 54)_ = 29.742, *p* < 0.01, Dunnett’s T3 test; *p* = 0.001, E2 vs OVX; *p* = 1.000, EA-L vs OVX; *p* = 0.054, EA-M vs OVX; *p* = 0.000, EA-H vs OVX for ALP; Fig 2A; F_(5, 54)_ = 6.777, *p* < 0.01, Dunnett’s T3 test; *p* = 0.011, E2 vs OVX; *p* = 1.000, EA-L vs OVX; *p* = 0.787, EA-M vs OVX; *p* = 0.017, EA-H vs OVX for OC; Fig 2B). In addition, the urinary DPD/Cr ratio (a bone resorption marker) was much more higher in OVX rats. Treatment with EA or E2 prevented this alteration in the OVX rats (F_(5, 54)_ = 15.713, *p* > 0.01, LSD test; *p* = 0.001, E2 vs OVX; *p* = 0.600, EA-L vs OVX; *p* = 0.000, EA-M vs OVX; *p* = 0.000, EA-H vs OVX; Fig 2C). The low dose of EA (1 mg/kg) had marginal effects on the above parameters.

**Table 2:** Effects of EA on biochemical parameters in serum and urine of OVX rats.

Group	S-Ca (mM)	S-P (mM)	U-Ca/Cr	U-P/Cr
Sham	2.61±0.04	3.3±0.2	0.39±0.01	3.7±0.2
OVX	2.56±0.06	3.4±0.1	1.05±0.06^##^	4.5±0.1^##^
E2	2.62±0.09	3.2±0.2	0.39±0.01**	4.0±0.2**
EA-L	2.60±0.03	3.4±0.1	1.02±0.04	4.5±0.1
EA-M	2.59±0.06	3.2±0.2	0.41±0.01**	4.3±0.1
EA-H	2.60±0.11	3.3±0.2	0.39±0.02**	4.2±0.2*

EA-L: EA (1 mg/kg); EA-M: EA (5 mg/kg); EA-H: EA (15 mg/kg); n = 10; n = 10

*p < 0.05

**p < 0.01 vs OVX

^##^ *p* < 0.01 vs sham.

### EA improves BMD and trabecular architecture in OVX rats

**Fig 3:**
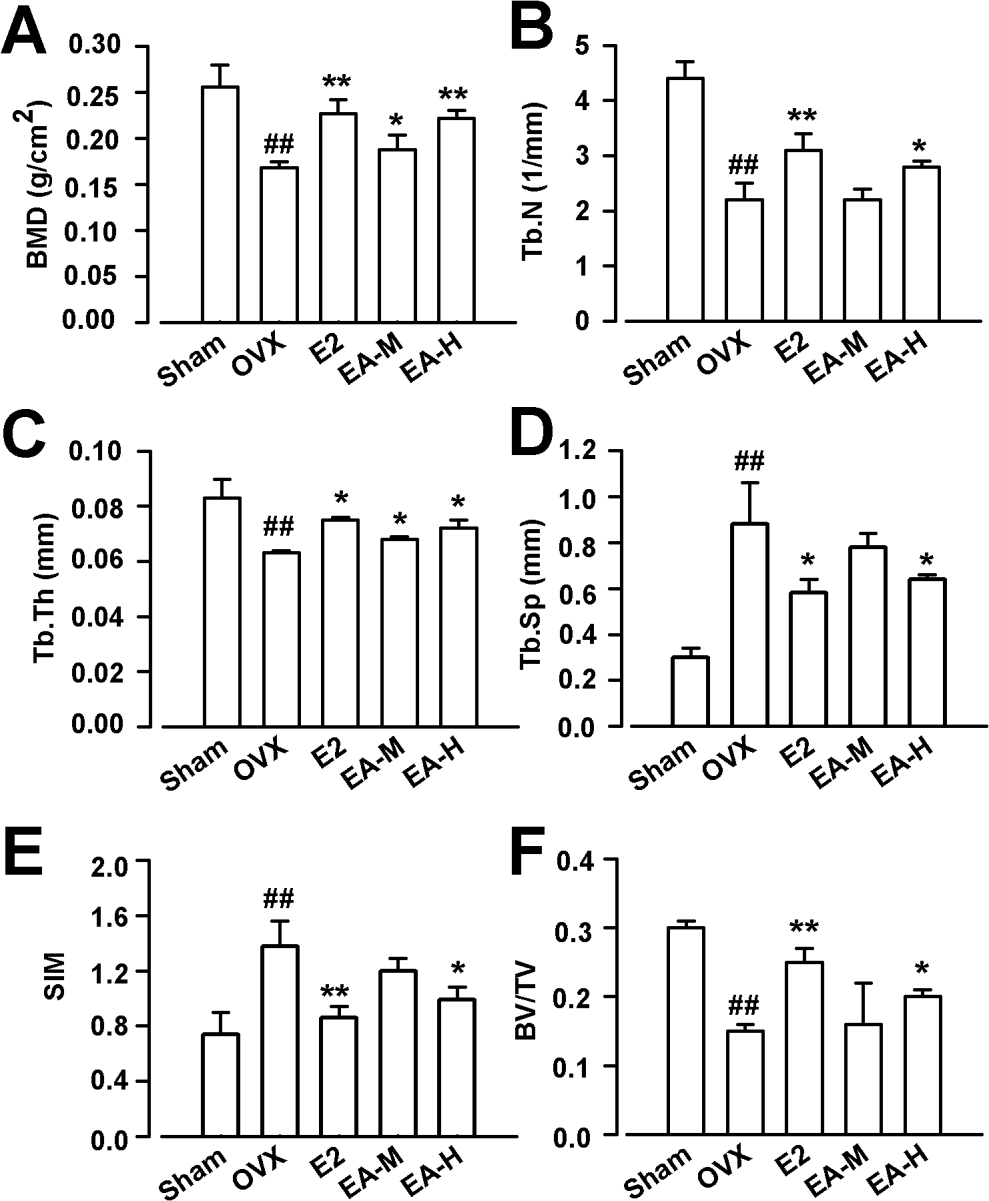
Effects of EA on the BMD and trabecular architecture. Effects of E2 and EA on BMD (A), trabecular number (Tb.N) (B), trabecular thickness (Tb.Th) (C), trabecular separation (Tb.Sp) (D), structure model index (SIM) (E), ratio of trabecular volume/bone total volume (F). E2 (25 μg/kg/day); EA-L (1 mg/kg/day); EA-M (5 mg/kg/day), EA-H (15 mg/kg/day). n = 10; *p < 0.05, **p < 0.01 vs OVX; ^##^ *p* < 0.01 vs Sham.

**Fig 4:**
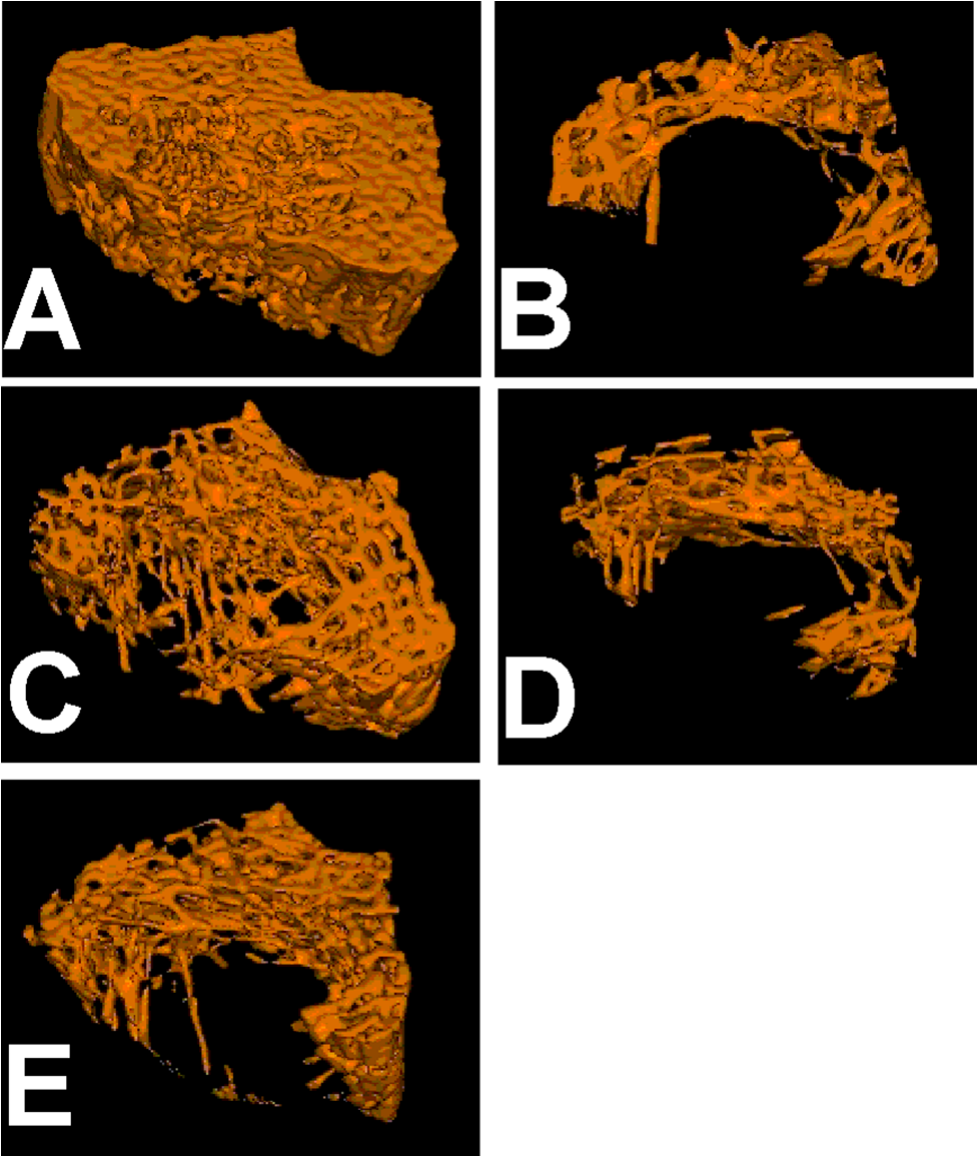
3-D architecture of trabecula bone. Representative micro-computed tomography samples within the distal metaphyseal femur region from Sham **(A)**, OVX **(B)**, E2(25 μg/kg/day) **(C)**, EA-M (5 mg/kg/day) **(D)**, and EA (15 mg/kg/day) treatment **(E)** rats.

Because the low dose of EA (1 mg/kg) had marginal effects on the bone metabolic biomarkers, Micro-computed tomography analysis was only performed in the rats treated with middle and high dose of EA. The BMD of the right femur was lower in OVX rats. Treatment with EA or E2 prevented this alteration in the OVX rats (F_(4, 45)_ = 10.831, *p* >0.01, LSD test; Fig 3). Further three-dimensional images of tibial metaphysis showed differences in the trabecular architecture among groups (Fig 4). Micro-computed tomography analysis revealed that levels of BV/TV, Tb.N, and Tb.Th were lower in OVX rats as compared with the shams. By contrast, SMI and Tb.Sp were higher. The EA (15 mg/kg) or E2 treatment prevented the OVX-induced deterioration of microstructure in trabecular, as shown by elevated levels of trabecular BV/TV, Tb.N, and Tb.Th in OVX rats (F_(4, 45)_ = 28.319, *p* < 0.01, Dunnett’s T3 test for BV/TV; F_(4, 45)_ = 30.417, *p* < 0.01, Dunnett’s T3 test for Tb.N; F_(4, 45)_ = 8.526, *p* > 0.01, LSD test for Tb.Th; Fig 3).

### EA reverses elevated levels of IL-1βand TNF-αin OVX rats

**Fig 5:**
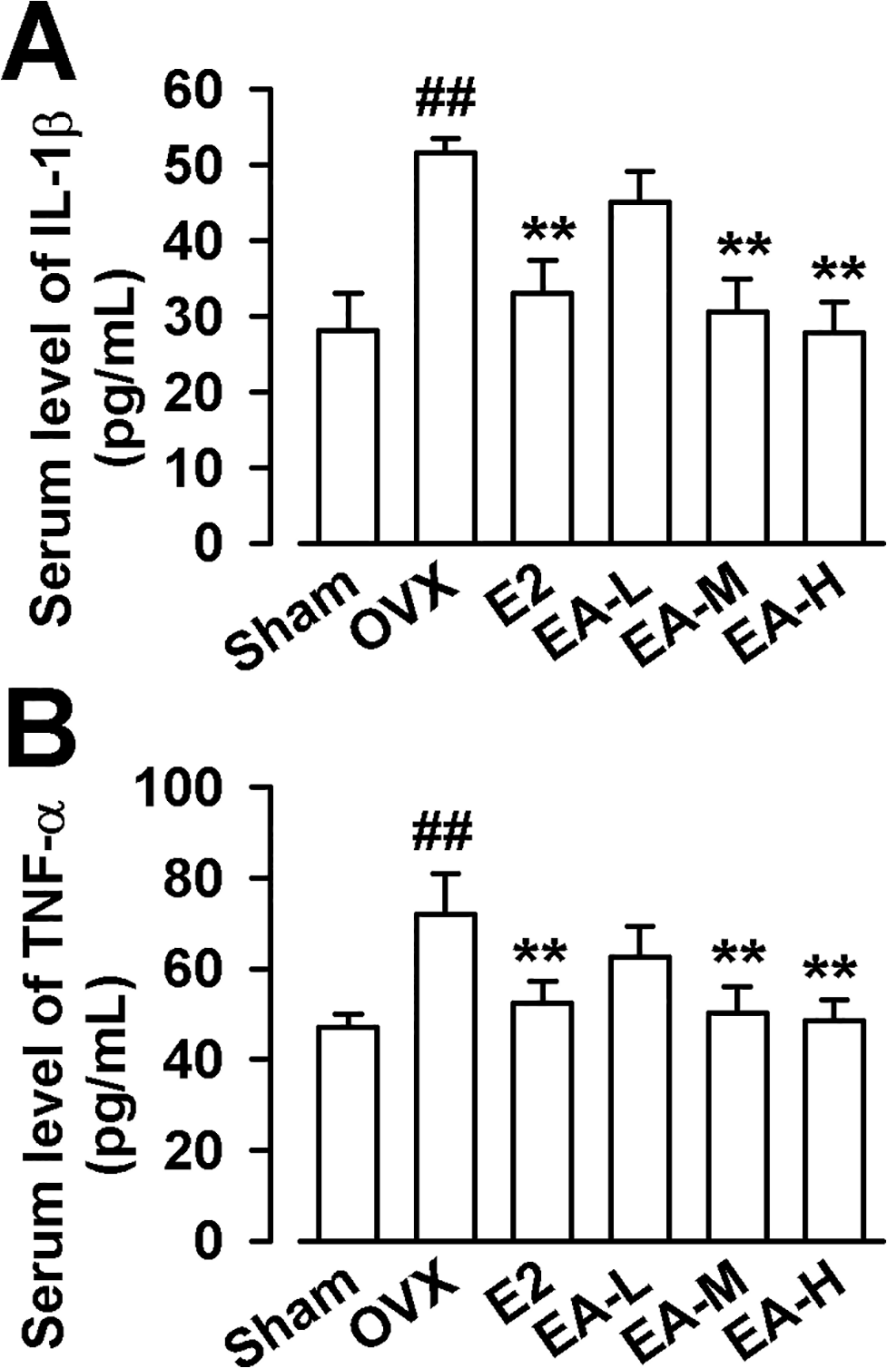
Effects of EA on IL-1β and TNF-α in rat serum. Effects of E2 and EA on serum IL-1β **(A)** and TNF-α **(B)**. E2 (25 μg/kg/day); EA-L (1 mg/kg/day); EA-M (5 mg/kg/day), EA-H (15 mg/kg/day). n = 10; *p < 0.05, **p < 0.01 vs OVX; ^##^ *p* < 0.01 vs Sham.

Since the activation of osteoclast function induced by OVX is largely dependent on the release of inflammatory mediators, it would be important to measure the serum levels of inflammatory cytokines in the rats treated with EA. As shown in Fig 5, the levels of IL-1β and TNF-α in OVX rats were hightest among groups. Treatment with E2 or EA prevented this alteration in the OVX rats (F_(5, 54)_ = 9.882, *p* < 0.01, Dunnett’s T3 test for IL-1β; F_(5, 54)_ = 40.883, *p* < 0.01, Dunnett’s T3 test for TNF-α; Fig 5).

## Discussion

The present study was designed to evaluate the potential effects of EA on osteoporosis induced by estrogen deficiency. Administration of EA was found to improve the maximum stress and Young’s modulus of femur in OVX rats. Micro-computed tomography analysis revealed that EA could improve the trabecular architecture, as shown by increasing the BV/TV, Tb.N, and Tb.Th in OVX rats. However, EA did not affect the body weight and uterine weight. Our data suggest that EA is a potential candidate or a leading compound for anti-osteoporosis.

Recently, significant attention has been focused on the possible antiosteoporosis role of terpenoids. Loquat leaves, which are rich in triterpone, have been shown to suppress the OVX-induced bone mineral density deterioration, and ursolic acid was isolated through bioactivity-guided fractionation [32]. A previous study confirmed that ursolic acid could stimulate osteoblast differentiation *in vitro* and induce new bone formation in a mouse calvarial bone formation model [14]. Series of ursolic acid derivatives targeted for bone anabolic were synthesized, wherein one was found to improve the bone microarchitecture without estrogenic side effects in OVX rats [33]. Oleanolic acid is the isomer of ursolic acid. Oleanolic acid and its glycosides have been reported to prevent bone loss [13]. Quinoxaline derivative of oleanolic acid could also inhibit the osteoclastic bone resorption and prevent the OVX-induced bone loss [15]. EA shares a similar structure with oleanolic acid.

In this study, EA was systematically investigated on its protection against OVX-induced bone loss for the first time. An ideal drug to treat bone fragility should improve the extrinsic biomechanical properties of bone, but at least not substantially impair the intrinsic properties [34]. Our results showed that EA at higher doses (5 mg/kg/day and 15 mg/kg/day) could prevent the OVX-induced decrease of elastic modulus and ultimate stress at the evaluated femur, but extrinsic biomechanical properties of the bone was not significantly improved. Recently, similar results were observed when treating the OVX rats with total lignans from the barks of *Eucommia ulmoides* [35,36].

The improvement of the intrinsic and extrinsic properties at the same time is difficult. For instance, fluoride can improve the bone mass [36], but fluoride incorporation into bone mineral reduce the intrinsic biomechanical properties [37]. Bone turnover is activated in osteoporosis, hence several bone metabolic biomarkers in serum or urine were evaluated. As expected, the OVX resulted in an increase in the bone turnover markers, as indicated by statistical significance in U-Ca, U-P, ALP, OC and DPD/Cr between OVX group and Sham group. However, previous studies report OC concentrations are not significantly higher in OVX group [26]. No significantly differences were observed concerning S-Ca and S-P in the study conducted by Zhang, while Shen found statistically higher levels of S-Ca in another study [25,26]. These slight differences among different studies might be related to the age of the animal used and the experimental period. EA showed osteoprotective effects in a dose-dependent manner. It had little effects at lower dose of 1 mg/kg/day, but prevented the alteration in serum ALP and OC, urinary Ca excretion, and DPD/Cr ratio at higher doses (5 mg/kg/day or 15 mg/kg/day). EA at higher doses prevented lower levels of BMD induced by OVX. BMD has been described as a major determinant of bone strength [38], however, it is insufficient to accurately predict the fracture risk and other parameters such as trabecular architecture should be considered in evaluating the impact of a treatment on bone strength [39]. Therefore, micro-computed tomography was used further to evaluate the morphology and structure of the trabecular bone. EA (15 mg/kg/day) treatment markedly reversed the alterations of trabecular BV/TV, Tb.N, Tb.Th, SMI and Tb.Sp, indicating the beneficial effects of EA on trabecular bone architecture.

Pro-infammatory cytokines (IL-1β, IL-6, IL-8 and TNF-α) play an important role in regulating bone resorption, while estrogen deficiency always leads to remarkable increase in pro-iflammatory cytokines [2]. In the study, the serum levels of IL-1β and TNF-α were much higher in OVX group, which is consistent with other studies [40,41]. EA has been shown to display an anti-inflammatory effect in different models of chronic inflammation in mice through the down-regulation of pro-inflammatory cytokines such as IL-1β, IL-18, TNF-α [16,17,18]. Therefore, we proposed that anti-osteoporosis effect of EA might be relevant with its anti-inflammatory effects. Our results demonstrated that EA (5 and 15 mg/kg) could significantly reverse the levels of IL-1β and TNF-α, but only EA (15 mg/kg) could improve the trabecular architecture. It is suggested that osteoprotective effects of EA partly contribute to its anti-inflammatory effects.

In conclusion, EA administration has the potential to improve the cancellous bone structure and biochemical properties in OVX rats without side effect on the uterus. It may serve as a new candidate or a leading compound for the development of antiosteoporosis drug. Although the preliminary results indicated the osteoprotective effects of EA partly contributed to its anti-inflammatory effects, the mechanisms of EA on osteoporosis are far to understand.

The limitation of this study is the age of animals used. Three-month-old female Sprague–Dawley rats are not old enough to mimic the osteoporosis in the aged animals. The three months of experimental period would not be considered as long-term for bone turnover. It might be possible that the effects of EA treatment for younger OVX rats exhibited more variance in present study.
